# Non-Pharmacological Treatment of Heart Failure—From Physical Activity to Electrical Therapies: A Literature Review

**DOI:** 10.3390/jcdd11040122

**Published:** 2024-04-17

**Authors:** Antonio Scarà, Zefferino Palamà, Antonio Gianluca Robles, Lorenzo-Lupo Dei, Alessio Borrelli, Federico Zanin, Leonardo Pignalosa, Silvio Romano, Luigi Sciarra

**Affiliations:** 1 San Carlo di Nancy Hospital—GVM, 00165 Roma, Italy; alessioborrelli@libero.it (A.B.); federicozanin89@gmail.com (F.Z.); leonardopignalosa@hotmail.com (L.P.); 2 Department of Life, Health and Environmental Sciences, University of L’Aquila, 67100 L’Aquila, Italy; zefferino.palama@icloud.com (Z.P.); antoniogianluca.robles@graduate.univaq.it (A.G.R.); lorenzolupo.dei@graduate.univaq.it (L.-L.D.); silvio.romano@univaq.it (S.R.); luigi.sciarra@univaq.it (L.S.); 3 Electrophysiology Unit “Casa di Cura Villa Verde”, 74121 Taranto, Italy; 4 Department of Cardiology, “L. Bonomo” Hospital, 76123 Andria, Italy

**Keywords:** heart failure, non-pharmacological treatment, catheter ablation, HF devices

## Abstract

Heart failure (HF) represents a significant global health challenge that is still responsible for increasing morbidity and mortality despite advancements in pharmacological treatments. This review investigates the effectiveness of non-pharmacological interventions in the management of HF, examining lifestyle measures, physical activity, and the role of some electrical therapies such as catheter ablation, cardiac resynchronization therapy (CRT), and cardiac contractility modulation (CCM). Structured exercise training is a cornerstone in this field, demonstrating terrific improvements in functional status, quality of life, and mortality risk reduction, particularly in patients with HF with reduced ejection fraction (HFrEF). Catheter ablation for atrial fibrillation, premature ventricular beats, and ventricular tachycardia aids in improving left ventricular function by reducing arrhythmic burden. CRT remains a key intervention for selected HF patients, helping achieve left ventricular reverse remodeling and improving symptoms. Additionally, the emerging therapy of CCM provides a novel opportunity for patients who do not meet CRT criteria or are non-responders. Integrating non-pharmacological interventions such as digital health alongside specific medications is key for optimizing outcomes in HF management. It is imperative to tailor approaches to individual patients in this diverse patient population to maximize benefits. Further research is warranted to improve treatment strategies and enhance patient outcomes in HF management.

## 1. Introduction

According to the definition adopted by current ESC guidelines [1], heart failure (HF) is not a single pathological diagnosis; rather, it is a complex clinical syndrome composed of cardinal symptoms (e.g., fatigue, breathlessness, and ankle swelling) and signs (e.g., elevated central venous pressure, pulmonary crackles, and peripheral edema). The onset of this syndrome can be related to different causes; in general, HF occurs when a structural or functional abnormality of the heart causes elevation of intracardiac pressures and/or a reduction in cardiac output at rest or during exercise.

HF has been categorized into three distinct phenotypes depending on the measured values of left ventricular ejection fraction (LVEF): HF with reduced EF (HFrEF) is defined when an EF ≤ 40% can be measured. HF with mildly reduced EF (HFmrEF) is defined by the presence of an EF between 41% and 49%. Finally, patients with symptoms and signs of HF, with evidence of structural and/or functional cardiac abnormalities and/or raised natriuretic peptides (NPs), and with an LVEF ≥ 50%, can be attributed to the last HF category: HF with preserved EF (HFpEF).

Despite the ejection fraction value, the most common classification used to describe the severity of HF is the New York Heart Association (NYHA) functional classification.

Heart failure (HF) represents a global public health issue affecting a very high number of individuals (more than 26 million) [1]. Despite progress in the theme of prevention and general health care, the prevalence of HF is steadily increasing worldwide; furthermore, it is expected to rise significantly due to the aging population phenomenon [2]. Despite significant advancements in treatment, HF remains a primary cause of hospitalizations with a high rate of one-year mortality rate (approximately 45%) among symptomatic patients [3].

Nowadays, a plethora of pharmacological treatments are approved and well established for the cure of HFrEF and HEmrEF. The combination of angiotensin-converting enzyme inhibitors (ACE-I) or an angiotensin receptor–neprilysin inhibitor (ARNI), a beta-blocker, and a mineralocorticoid receptor antagonist (MRA) is established as the cornerstone treatment for these HF-subtype patients (except in those cases when these drugs are contraindicated or not tolerated) [4,5,6]. The sodium–glucose co-transporter2 (SGLT2) inhibitors dapagliflozin and empagliflozin added to the triad (ACE-I/ARNI/beta blocker/MRA) lead to a reduction in the risk of CV death and worsening HF in patients with HFrEF [7,8]. Recently, the ESC’s focus update for the diagnosis and treatment of acute and chronic heart failure [9], based on the EMPEROR-Preserved [10] and DELIVER trial [11] acquisitions, recommended the use of SGLT2i drugs in class I level of evidence A for patients with HFpEF. 

Despite this important pharmacopeia, a consistent number of patients remain symptomatic for HF. The objective of the present review is to explore the effects of non-pharmacological treatments such as lifestyle modifications, physical activity/continuous training, or electrical therapies (cardiac ablations, resynchronization therapy/physiological pacing, cardiac contractility modulation) in the setting of HF.

Treating heart failure through surgical strategies represents a cornerstone of the management of this pathology. For example, coronary reperfusion or surgical interventions (coronary artery bypass graft surgery) for ischemic heart disease, surgical or percutaneous mitral or aortic valve procedures for severe valvulopathies, or the implantation of ventricular assist devices and heart transplantation are fundamental elements of the therapeutic arsenal that can bring significant benefit to the quality of life of patients. These often represent a true lifesaver. However, the discussion of these techniques has been well-executed elsewhere and is beyond the aim of this review.

## 2. Lifestyle Measures

As recognized by ESC 2021 guidelines for the diagnosis and treatment of heart failure, adequate patient self-care is crucial for effective heart failure (HF) management, contributing significantly to improved quality of life (QOL), lower hospital readmission rates, and reduced mortality among HF patients [1]. 

Patient education on the mechanisms of heart failure and lifestyle changes that might be beneficial plays a crucial role in enhancing self-care skills with tailored education based on scientific evidence or expert opinion being fundamental. General approaches to patient education include providing information in various formats to accommodate educational grade and health literacy levels, employing strategies like ‘ask–tell–ask’, ‘teach back’, or motivational interviewing, and addressing communication barriers.

Regarding dietary habits, ESC guidelines emphasize the need for a “healthy diet” with nutrition having to be adapted to the specific needs of every single patient. Nutrition for weight loss is recommended in overweight and obese heart failure patients, and nutritional guidance to restrict calories intake should be recommended. However, it is known that in advanced heart failure patients (class C and D), attention should be paid to the so-called “obesity paradox” [12]. This kind of patient should be counseled to adapt their diet to reduce weight loss and in doing so avoid sarcopenia and cardiac cachexia, which are all factors associated with heart failure that are known to worsen prognosis [13].

Independently of the body fat percentage, particular attention should be paid to micronutrient deficiencies (i.e., iron) that should be avoided and eventually treated [14]. Although evidence for the specific benefits of sodium restriction in HF patients is limited, some recent clinical trials show promising results in improving NYHA functional class and edema, particularly in more congested patients [15,16,17,18].

ESC 2021 guidelines state that sodium restriction (<5 g/day) is advisable for patients with HF to alleviate congestive symptoms. However, achieving and maintaining sodium restriction can be challenging and may lead to poor dietary quality without proper guidance. 

Furthermore, lifestyle interventions encompass more than just diet. 

Addressing smoking cessation [19], stress management [20], and alcohol intake is essential [21]. In particular, stress management interventions targeting psychological stress and other psychosocial factors associated with heart failure (HF) morbidity and mortality demonstrated short-term improvements in anxiety, depressive symptoms, quality of life, and even exercise capacity [20]. 

Patients with HF should receive specific education and support in a multidisciplinary approach, involving cardiologists, nurses, pharmacists, dieticians, mental health clinicians, and social workers [1]. 

On top of this kind of attention by the clinician to patient care, exercise rehabilitation, as recommended by both ESC and AHA/ACC guidelines, consistently demonstrates benefits in improving exercise tolerance, QOL, and reducing all-cause and HF hospitalizations [1]. 

Lifestyle modifications are integral components of HF management from the patient’s perspective. These measures, when combined with tailored education and exercise rehabilitation, can significantly improve patient outcomes. 

## 3. Physical Activity/Continuous Training

Current American College of Cardiology/American Heart Association guidelines for heart failure (HF) management include a class 1 recommendation (Level of Evidence A) for exercise training in HF patients [22]. Quality of life (QoL) serves as an established outcome and prognostic factor in HF; optimal QoL significantly affects survival outcomes [23]. For clinically stable chronic HF with reduced ejection fraction (HFrEF) patients, regular physical activity or exercise training is indicated to enhance functional status, QoL, and mortality risk [13]. Several studies confirm the effectiveness of aerobic workouts in HFrEF patients [24]. Aerobic exercise training is a recognized non-pharmacological approach for improving HF’s pathophysiological, clinical, and prognostic aspects. Precision in prescribing appropriate exercise intensity is crucial for maximizing benefits and minimizing risks. Given HFrEF’s progressive nature and significant breathlessness, interval exercise is typically preferred [25]. The existing literature consistently favors interval training over continuous training, particularly at low or moderate intensity, for this patient population [26].

Recent evidence suggests moderate continuous training (HICT) may offer more benefits than high-intensity interval training (HIIT) [27]. However, recent findings indicate that patients tolerate higher intensities of continuous training well and experience greater cardiovascular improvements compared to lower intensities [28]. Specifically, studies on humans show that HICT increases cardiorespiratory fitness (VO2max) and cardiac output [29]. A meta-analysis of 56 articles found a direct correlation between VO2max, endothelial function, and smooth muscle function, indicating that HICT’s impact on cardiac remodeling is linked to VOmax [30]. Compared to lower intensities, HICT induces the most significant change in V02max [31,32]. 

According to ESC guidelines, a supervised, exercise-based, cardiac rehabilitation program should be considered in patients with more severe disease, frailty, or with comorbidities (class IIa LoE b) [1].

Transitioning from HFrEF to heart failure with preserved ejection fraction (HFpEF), supervised exercise training (SET) safety is consistently demonstrated in selected middle-aged and older chronic, stable HFpEF patients. A meta-analysis of 276 patients from six randomized trials reported no exercise-related major adverse events [33]. In the only trial comparing SET effects in older chronic HFpEF and HFrEF patients directly, there was a significant peak VO2 improvement in HFpEF but not HFrEF at 4 months of follow-up [34]. Overall, available data indicate that SET’s potential to improve exercise capacity in chronic HFpEF patients is at least equal to, and possibly greater than, that seen in chronic HFrEF patients [35].

## 4. Catheter Ablation

Among the different treatments available for HF, there is also space for catheter ablation (CA) in some clinical settings. In particular, CA may be employed in the following contexts: (1) atrial fibrillation and (2) PVCs induced or worsened cardiomyopathy. In the next two sections, we will discuss the application of CA in the aforementioned fields.

### 4.1. Atrial Fibrillation

CA application on patients with atrial fibrillation and HF is the object of different randomized trials and has clear recommendations according to the last European and American guidelines on AF management [36,37]. In particular, we should recognize different scenarios in which AF interplays with HF and vice versa. AF may be the cause of HFrEF in patients in whom AF may induce a—generally—reversible left ventricular dysfunction named tachycardiomiopathy because it is due to a long period of high ventricular rates. Conversely, AF may be an epiphenomenon of HF and so it may belong to the long list of conditions related to HF. 

In the first scenario, rhythm control—and specifically, achieved by CA—has a great probability of restoring left ventricular dysfunction following sinus rhythm restoration. However, in some cases, when doubt can subsist about the pathogenesis of the ventricular dysfunction, it could be reasonable to perform preliminary atrial cardioversion to evaluate left ejection fraction in sinus rhythm, even if this strategy can be limited by a high probability of arrhythmia relapse despite antiarrhythmic drugs [38,39].

In the second scenario, CA ablation has the aim to restore sinus rhythm or significantly reduce the arrhythmic burden. It also has the role of improving QoL, reducing hospitalization, easing left ventricle reverse remodeling and thus improving LVEF, and reducing thromboembolic complications [40]. 

Taken together, all of these aim to reduce HF and AF-related disability and improve mortality [41]. According to the last European and American guidelines of AF management, given the positive results from the randomized trial CASTLE-AF [36,37,38], CA of AF in patients with HfrEF has gained a class I level of evidence recommendation. Interestingly, a CABANA trial sub-analysis showed outcome improvement using CA for AF rather than medical therapy in patients with HFpHF [39,40]. At this point, the concept of personalized therapy should be reiterated: not all HF patients with AF should undergo CA, but only a selected population (preferably those patients meeting CASTLE-AF inclusion criteria, representing about 10% of all HF population) with symptomatic AF and/or reduced QoL from AF [38]. Moreover, it should be underlined that the superiority of CA over antiarrhythmic drugs (AADs) for rhythm control is more relevant in the context of HFrEF patients in whom class I AADS are not allowed and class III may have significative tolerance issues or side effects [36].

Up until now, we referred only to paroxysmal or persistent AF forms, which are candidates for pulmonary vein isolation (PVI) and in selected cases also substrate modification (for persistent ones). Long-standing or permanent AF also deserves attention regarding ablation. These categories of patients—and above all permanent AF—do not represent the ideal category to undergo PVI—and substrate modification—given the already severe established atrial cardiomyopathy [41]. 

In cases where optimal rate control with medication is not achieved or not tolerated, patients become good candidates for the ablate and pace strategy, which is entering a new era fueled by the enthusiasm and promising results from physiological pacing [42,43].

### 4.2. PVCs

A subset of dilated or hypokinetic non-dilated cardiomyopathy may be due to a high burden of PVCs. In particular, a high PVC burden may be the primary movens of this kind of—generally reversible—left ventricular dysfunction. Also, it may determine a further worsening of the LVEF in patients previously suffering from a structural heart disease (with or without dysfunction). The former clinical scenario is suspected when at least 10% PVC burden is present together with LV dysfunction, and in this case, PVCs ablation has been recognized as class IC of recommendation by the last European guidelines on the management of ventricular arrhythmias [44]. Conversely, in the latter scenario, the class of recommendation for VAs CA is IIA [44]. CA is highly effective with up to 75–90% efficacy but, as easily understandable, it is affected by the focus of origin of the PVC (outflow tracts and fascicular origins have the best results compared to LV summit or epicardial ones), the number of PVC morphologies (single or predominately one morphology vs. polymorphic) and the presence and extent of LGE (which configure the presence of an underlying heart disease influencing patient’s prognosis) [44].

Finally, PVC ablation has a class IIA recommendation in patients in whom appropriate biventricular pacing is not achieved due to the high burden of VAs [44].

### 4.3. Ventricular Tachycardia

Ventricular tachycardia prevention is a key factor in reducing the risk of arrhythmic death in heart failure patients. For patients with ischemic cardiomyopathy and an ICD, both catheter ablation and antiarrhythmic drugs have been shown to decrease the occurrence of ICD shocks in randomized trials [45,46,47]. 

Ablation appears to be a superior option compared to escalating antiarrhythmic drugs (AADs) in patients experiencing ventricular tachycardia (VT) recurrences despite the prior use of amiodarone. However, there were no randomized studies comparing the efficacy and safety of both treatments in AAD-naïve patients [48]. 

For this specific topic, the SURVIVE-VT trial evaluated the effectiveness and safety of catheter ablation compared to AADs as the initial treatment option for ICD-carrying patients experiencing symptomatic VTs. What it found was that compared to AAD, catheter ablation led to a decrease in the composite endpoint of cardiovascular death, appropriate ICD shock, hospitalization due to heart failure, or severe treatment-related complications [49]. 

Moreover, whether ventricular tachycardia ablation might provide any prognostic benefit to heart failure patients already carrying an ICD, and the correct timing for such a procedure in this population has also been debated in recent years. This was the aim of the PARTITA trial. It showed that in a population of ischemic and non-ischemic dilated cardiomyopathy patients, performing ventricular tachycardia ablation after the first appropriate shock was associated with a reduced risk of combined death or worsening heart failure hospitalization along with lower mortality rates and fewer ICD shocks. These findings lend support to the idea of considering ventricular tachycardia ablation following the initial ICD shock [50]. 

Further exploration of this was carried out in the PAUSE-SCD trial to investigate the correct timing of catheter ablation in HF patients not already carrying an ICD. A population of 121 patients with various cases of heart failure, symptomatic ventricular tachycardia, and indication for ICD implantation were randomized (1:1) to ablation plus an ICD versus conventional medical therapy plus an ICD. Performing early catheter ablation at the time of ICD implantation resulted in a significant reduction in the composite primary outcome of VT recurrence, cardiovascular hospitalization, or death. This reduction was primarily driven by a decrease in the need for ICD therapies [51]. 

## 5. Resynchronization Therapy/Physiological Pacing

Cardiac resynchronization therapy (CRT) stands as the principal non-pharmacological treatment for moderate to severe heart failure with its effectiveness supported by numerous clinical trials [52,53]. CRT has been shown to alleviate symptoms and enhance left ventricular function in numerous heart failure patients with left ventricular systolic dysfunction and cardiac dyssynchrony. Recommendations for CRT are derived from the findings of major randomized clinical trials (RCTs) for CRT, most of which have focused on approximately 60% of heart failure patients with reduced ejection fraction who are in sinus rhythm. CRT, alongside guideline-directed medical therapy, is specifically recommended for a defined subset of the heart failure patient population, predominantly those with symptomatic heart failure in sinus rhythm, a reduced LVEF, and a QRS duration of 130 milliseconds or greater. 

Other potential candidates for CRT are NYHA class III or IV HF patients in AF with reduced LVEF and QRS ≥ 130 ms, provided there is a strategy for biventricular capture or anticipated return to sinus rhythm.

Additionally, CRT may be considered occasionally as an upgrade from a conventional pacemaker or an ICD in HFrEF patients who develop worsening HF with a high rate of ventricular pacing [54]. In this setting, apical right ventricular pacing has been described to impair left ventricular ejection fraction; when this scenario occurs, an upgrade to CRT can lead to a significant improvement of left ventricular performance and a consistent reduction in the number of hospitalizations for HF, similarly to native CRT strategy [55].

Not all patients respond positively to CRT. Specific traits predict ventricular volume reduction (reverse remodeling) and better outcomes. QRS width, a criterion in all trials, predicts CRT response. QRS morphology is also linked to favorable CRT outcomes. Not all patients respond positively to CRT. Specific traits predict ventricular volume reduction (reverse remodeling) and better outcomes. QRS width, a criterion in all trials, predicts CRT response. QRS morphology is also linked to favorable CRT outcomes. LBBB morphology is more likely to respond favorably, whereas there is less certainty about patients with non-LBBB morphology [56,57]. In daily clinical practice, even in the best settings, the response to CRT is far from 100%. Attempts have been made to identify echocardiographic parameters that could somehow overcome the mere ECG criterion but without obtaining satisfactory results. Even the echocardiographic criterion alone has proven to be harmful for patients undergoing CRT implantation. Nowadays, the width of the QRS, its morphology, the electrical axis of the LBBB, and underlying heart disease still represent the best predictors of success of this therapy [58,59,60,61]. 

Beyond the choice of the ideal candidate for CRT, there are also technical procedural aspects to consider that may influence the success of this therapy: the anatomy of the branches of the coronary sinus, the stimulation thresholds of any fibrotic tissues, etc. For this reason, the use of better imaging techniques during implantation (for example with the use of non-fluoroscopic mapping systems) [62,63] and of different algorithms and stimulation modalities (for example with multipolar stimulation) can improve the outcome of this therapy [64,65].

**Figure 1:**
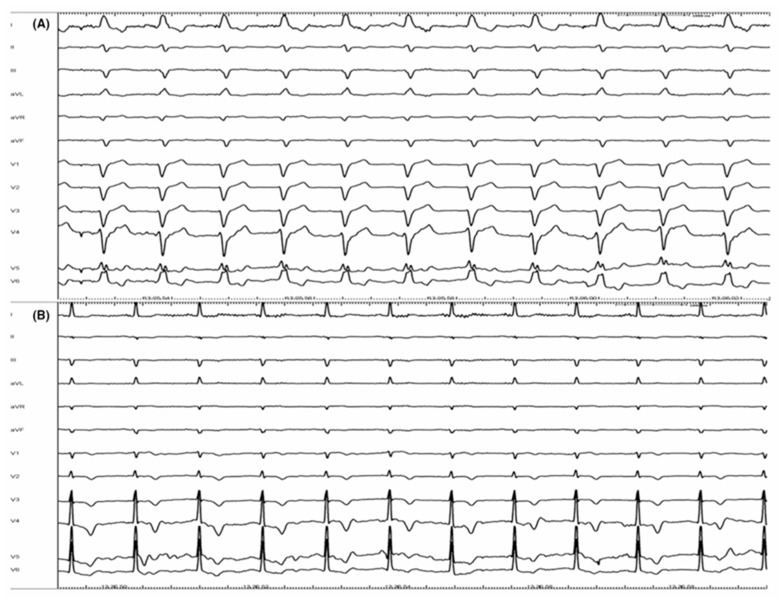
Correction of complete left bundle branch block (CLBBB)**:** panel (**A**) shows a basal ECG with CLBBB. Panel (**B**) represents the ECG from the same patient at the end of procedure. CLBBB has been completely corrected by a selective His bundle pacing (HBP) (with permission by Scarà et al. [61]).

To overcome the CRT’s limitations, physiological pacing (of the His bundle or the left branch area) has established itself as a support for CRT. His bundle pacing, in some cases, can itself correct the left bundle branch block [66] (Figure 1). In the HOT-CRT trial, His–Purkinje conduction system pacing was used in patients with heart failure, and a coronary sinus (CS) lead was added only if His pacing resulted in incomplete electrical resynchronization. This strategy resulted in a greater change in LVEF compared with the CRT approach only [67].

Unfortunately, His pacing is burdened by a high rate of implant revisions. For this reason, in recent years, left bundle branch stimulation (LBBP) has become established, which has a better acute success rate and a low complications rate [68].

Although outlined by the guidelines as an alternative to CRT in case of failure, non-randomized controlled studies are demonstrating greater efficacy of LBBP to have better electromechanical resynchronization, higher clinical and echocardiographic response, and an especially higher rate of super-response than conventional CRT in patients with LVEF ≤ 35% and LBBB with HF [69,70,71].

## 6. Cardiac Contractility Modulation (CCM)

Cardiac contractility modulation (CCM) represents a new therapeutic chance for those patients suffering from symptomatic heart failure (NYHA class III–IV) who do not match the criteria for CRT or who are non-responders to CRT therapy. The last ESC guidelines (2021) classified this technology as “devices under evaluation”. However, in patients with NYHA class III–V HF, with an LVEF ≤ 25% to ≤45% and QRS duration < 130 ms, CCM implantation was associated with an improvement in exercise tolerance and QOL [72].

### 6.1. Device and Implantation Procedure

**Figure 2:**
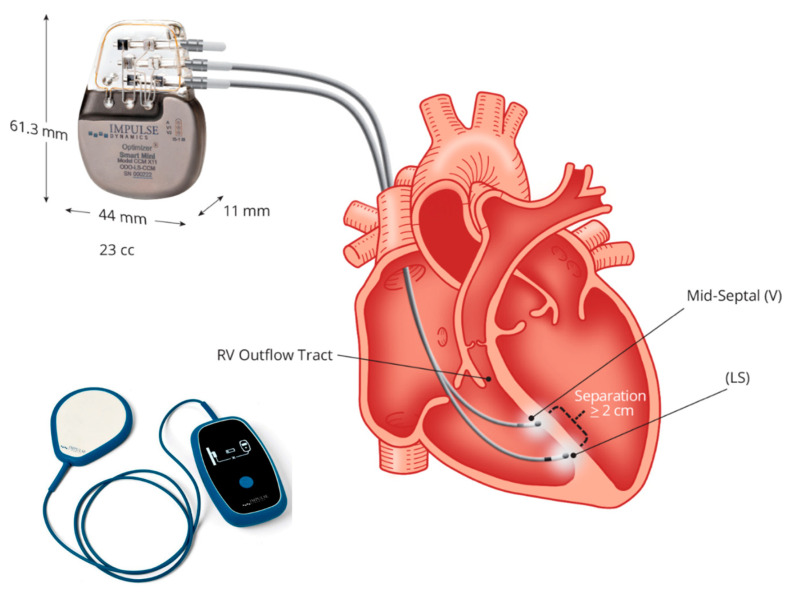
The Optimizer system: figure shows the implantable pulse generator with a rechargeable battery and the battery charger. Two ventricular pacing screw-in leads (atrial lead is non-mandatory), implanted to the right septum allow energy delivery from the pulse generator for cardiac contractility modulation (CCM). Courtesy of Impulse Dynamic.

Up-to-date CCM is feasible using a single technology: the Optimizer system. This is a singular technology consisting of an implantable pulse generator equipped with a rechargeable battery, two ventricular pacing screw-in leads (an optional atrial lead), an implantable pulse generator programmer, and a battery charger (Figure 2). To summarize the device and its implantation procedure, an optional atrial lead may be used for sensing, which is placed akin to standard pacemakers and defibrillators. Two ventricular leads, serving for sensing local electrical activity and delivering CCM signals, are positioned on the right ventricular septum and programmed to deliver CCM signals during five 1-h periods evenly spaced throughout the day [73,74,75].

### 6.2. Mechanism of Action

CCM signals are non-excitatory electrical pulses administered during the cardiac absolute refractory period, enhancing cardiac muscle contraction strength [68]. This mechanical effect seems linked to restoring impaired cellular functions in heart failure. Preclinical studies have demonstrated a swift positive inotropic effect of CCM, which is potentially mediated by modulating cardiomyocyte Ca^(2+)^ fluxes and altering cardiac phospholamban phosphorylation. In translational and clinical studies, utilizing double biphasic voltage pulses to the right ventricular aspect of the interventricular septum, sustained positive effects on cardiac reverse remodeling and contractility have been observed over time [73,74,75,76,77].

### 6.3. Limitations and Future Perspectives

The number of intravascular electrocatheters can represent a possible limitation for the implantation of this device in some cases: for example, in those patients who received a previous CRT device with an inadequate clinical response or when an additional implantable automatic defibrillator is required (in primary prevention due to reduced ejection fraction or in secondary prevention). To date, however, apart from these concerns, there are no significant clinical side effects described also in these scenarios. Anyway, in a very short time, it will be reasonable implantable a device (Optimizer Integra) combining both CCM and ICD technologies.

## 7. Digital Health Applications in Heart Failure

The term “digital health” encompasses the use of technology in medicine and other healthcare fields to manage illnesses, mitigate health risks, and promote overall well-being. This includes wearable devices, mobile health applications, telehealth services, health information technology systems, and telemedicine. 

Teleconsultations are remote medical exchanges (via video or phone calls), allowing assessment and management without in-person visits. They can be “provider to provider” or “provider to patient”. The first was evaluated for the first time in the IMPLEMENT-HF study showing that a virtual team, even in a non-cardiovascular hospital setting, could help clinicians dialogue and was associated with improved heart failure therapeutic optimization [78,79].

“Provider-to-patient teleconsultations” showed suboptimal efficacy in obtaining guideline-directed medical therapy (GDMT) dosages with telephone teleconsultations having the worst results [80,81,82].

Telemonitoring is the remote tracking of vital signs and health data using technology such as wearable devices and sensors. This information is transmitted to health-care professionals for assessment, facilitating healthcare management and interventions. Telemonitoring can be utilized to optimize pharmacological treatment and align it with GDMT. In this context, randomized clinical trials have been conducted to assess the potential utility of such monitoring in tracking therapy compliance and ensuring appropriate treatment modifications. For instance, an RCT by Antonicelli et al. demonstrated that telemonitoring was associated with a more appropriate use of ß-blockers in patients with heart failure with reduced ejection fraction (HFrEF) [83]. Similarly, in 2020, Artanian et al. illustrated that telemonitoring facilitated achieving optimal pharmacological therapy in a shorter time compared to the non-telemonitored control group [84,85]. 

These findings were further supported by studies conducted by Brahmbhatt et al. and Giordano et al. [86,87]. 

Significantly, in the VITAL-HF study, where automated alerts were triggered based on vital signs and laboratory data, patients reported that the intervention addressed concerns related to daily uncertainties, provided them with a sense of security, and empowered them to comprehend decision making concerning GDMT [88]. 

Recently, a randomized controlled trial (RCT) conducted by Romero et al. demonstrated that patients with HFrEF who utilized wireless devices to transmit daily heart rate, blood pressure, and weight data exhibited significantly closer adherence to GDMT dosages at the 6-month follow-up [89]. 

Telemonitoring can also be performed via cardiac implantable electronic devices. The most common way to achieve this is using data derived from pacemakers and implantable cardiac defibrillators. While primarily utilized for arrhythmia monitoring, they also play a role in HF management. The 2022 MANAGE-HF study showed that the “decongestion alert” via these devices helped in increasing diuretic therapy in 74% of the alerts [90]. Regarding β-blockers therapy, however, a post hoc analysis of the EFFECT study showed that in a “real-world” setting, there was no association between remote ICD data monitoring and the achieved dose of β-blockers [91]. 

Other useful devices are pulmonary artery pressure monitors, which can detect worsening congestion. Adamson et al.’s post hoc analysis of the CHAMPION RCT demonstrated that pressure remote control via PAP monitors facilitated more precise adjustments in diuretic therapy compared to the control group, which relied on symptoms and daily weights [92]. These findings were corroborated in the MONITOR-HF RCT where hemodynamic monitoring substantially improved quality of life and reduced heart failure hospitalizations [93]. 

While these initial findings are promising, there remains a necessity for further studies to determine the safety and effectiveness of digital solutions in optimizing GDMT for patients with heart failure.

Additionally, the integration of artificial intelligence solutions may aid in managing larger datasets and reducing the workload of medical professionals.

## 8. Conclusions

Heart failure (HF) still represents a significant public health concern with increasing prevalence, morbidity, and mortality rates despite advances in pharmacological therapies. Our review explored the role of non-pharmacological interventions in the management of HF, such as physical activity, catheter ablation, cardiac resynchronization therapy (CRT), and cardiac contractility modulation (CCM).

Physical activity has emerged as a vital component of HF management and proved to be beneficial in enhancing functional status, quality of life, and mortality risk reduction. This is particularly true for patients with HF with reduced ejection fraction (HFrEF). To date, the optimal intensity and type of exercise remain unclear, but the authors agree that individualized exercise prescription tailored to patients’ capabilities is essential.

It is frequent for heart failure to evolve in AF. In such a setting, catheter ablation has positive outcomes in improving left ventricular function and enhancing quality of life in selected HF patients. Furthermore, catheter ablation for premature ventricular contractions (PVCs) has proven to improve left ventricular function and overall prognosis.

CRT, in selected cases, continues to be a cornerstone non-pharmacological therapy for HF, helping to achieve left ventricular reverse remodeling, symptomatic relief, and mortality reduction. The identification of optimal candidates, by considering factors such as QRS morphology and width, is pivotal. 

The emerging therapy of CCM presents a promising opportunity for patients with symptomatic HF who do not meet CRT criteria or are CRT non-responders. While further research is needed to elucidate its long-term efficacy and safety profile, initial studies suggest potential benefits in improving exercise tolerance and quality of life.

In conclusion, our review highlights the importance of integrating non-pharmacological interventions into the comprehensive management of HF alongside pharmacotherapy.

Tailored approaches that consider individual patient characteristics and preferences are essential for optimizing outcomes. Further research and clinical experience are warranted to improve the treatment strategies and outcomes of heart failure patients.
